# A Spectral Graph Regression Model for Learning Brain Connectivity of Alzheimer’s Disease

**DOI:** 10.1371/journal.pone.0128136

**Published:** 2015-05-29

**Authors:** Chenhui Hu, Lin Cheng, Jorge Sepulcre, Keith A. Johnson, Georges E. Fakhri, Yue M. Lu, Quanzheng Li

**Affiliations:** 1 School of Engineering and Applied Sciences, Harvard University, Cambridge, Massachusetts, USA; 2 Department of Engineering, Trinity College, Hartford, Connecticut, USA; 3 Division of Nuclear Medicine and Molecular Imaging, Department of Radiology, Massachusetts General Hospital, Boston, Massachusetts, USA; 4 Center for Advanced Medical Imaging Science, Division of Nuclear Medicine and Molecular Imaging, Department of Radiology, Massachusetts General Hospital, Boston, Massachusetts, USA; National Laboratory of Pattern Recognition, CHINA

## Abstract

Understanding network features of brain pathology is essential to reveal underpinnings of neurodegenerative diseases. In this paper, we introduce a novel graph regression model (GRM) for learning structural brain connectivity of Alzheimer's disease (AD) measured by amyloid-*β* deposits. The proposed GRM regards ^11^C-labeled Pittsburgh Compound-B (PiB) positron emission tomography (PET) imaging data as smooth signals defined on an unknown graph. This graph is then estimated through an optimization framework, which fits the graph to the data with an adjustable level of uniformity of the connection weights. Under the assumed data model, results based on simulated data illustrate that our approach can accurately reconstruct the underlying network, often with better reconstruction than those obtained by both sample correlation and ℓ1-regularized partial correlation estimation. Evaluations performed upon PiB-PET imaging data of 30 AD and 40 elderly normal control (NC) subjects demonstrate that the connectivity patterns revealed by the GRM are easy to interpret and consistent with known pathology. Moreover, the hubs of the reconstructed networks match the cortical hubs given by functional MRI. The discriminative network features including both global connectivity measurements and degree statistics of specific nodes discovered from the AD and NC amyloid-beta networks provide new potential biomarkers for preclinical and clinical AD.

## Introduction

Alzheimer’s disease (AD) is the most common form of dementia, affecting approximately 10% of individuals of age 65. The prevalence increases quickly up to age 80, above which the incidence rate exceeds 40%. Today, the estimated number of AD patients in the US alone is 5.4 million, meaning that about one in eight older Americans has AD. By 2050, the AD prevalence in the US is anticipated to be 11 million to 16 million, with one new case every 33 seconds, or almost a million per year [1]. Although AD was first identified over a hundred years ago, current treatments only help relieve symptoms of the disease and there is still no cure. Monitoring preclinical pathological variations in the brain is probably a key to predicting the development of AD [2].

The advancements of neuroimaging techniques provide a promising tool for the early detection of AD. Changes in brain histopathology, and consequently in its structure and function, are known to precede the clinical manifestations of the disease by many years [3]. These modifications can be visualized *in vivo* using brain imaging modalities. Computerized tomography (CT) and magnetic resonance (MR) can be used to visualize internal structures of the body in detail; whereas functional MRI (fMRI) and positron emission tomography (PET) with tracers such as fluorodeoxyglucose (FDG), enable the measurement of neural-related activities [2]. More recently, Pittsburgh Compound-B (PiB) has been employed as a tracer for monitoring fibrillar amyloid-*β* (A*β*), the principal constituent of AD senile plaques deposited in brains of AD patients. Since the accumulation of A*β* usually occurs much earlier than the expression of AD symptoms, PiB-PET provides sensitive and consistent biomarkers in the preclinical stage of the disease [4–6].

It has been revealed that AD neurodegeneration targets functional brain networks [7]. The network nature of AD may explain why we have not achieved satisfactory classification rates by only comparing region-wise differences of neuroimaging data. Regarding the human brain as a network motivates a paradigm shift from studying isolated brain areas to understanding their mutual connections. Since many brain diseases such as AD are shown to be tightly associated with alternations in functional brain networks [8–10], various statistical methods have been adopted to infer the latent structural and functional connectivity from neuroimaging data. One of the mainstream techniques is correlation analysis, which estimates the correlation matrix by computing the sample correlation of the data. However, this method does not rule out the effect of other brain regions when evaluating pairwise correlations. Another approach is to use partial correlation, *i.e.*, normalized version of the inverse covariance matrix [11], which is often ill-conditioned, due to limited amount of samples in practice. Additional regularizations such as network sparsity [12–15] are usually imposed to overcome this problem. One can also estimate a sparse correlation matrix by a linear regression model with ℓ_1_-norm penalty [16], which solves a modified optimization problem compared with that in the aforementioned works. Other techniques to estimate a meaningful structural and functional network span from *multivariate statistical methods*, including *e.g.*, principle component analysis [17], independent component analysis [18], to *dynamic models*, including *e.g.*, dynamic causal models [19] and Granger causality [20]. Nevertheless, the first set of approaches, *i.e.*, the multivariate statistical models, have potential difficulties in mapping the results to biological entities; the latter, *i.e.*, the dynamic models, are either computationally demanding or dependent on the linearity of the data [11, 20].

In this paper, we propose a novel framework for learning brain structural connectivity and verify its effectiveness. The fundamental assumption of our graph regression model (GRM) is that the observed data are smooth signals on a deterministic graph. As a consequence, fewer samples are required to estimate the graph [21, 22]. We formulate the graph regression as an optimization problem with an adjustable level of uniformity of the connection weights in the objective function and a set of linear constraints. Since the GRM does not rely on a specific probability distribution of the data (*e.g.*, multivariate Gaussian distribution), it is more flexible than the previous statistical methods. We verify the model by utilizing both synthetic and real data. In the simulated analysis, we demonstrate that our approach can achieve a very accurate reconstruction of the true network, yielding better results than those obtained by sample correlation method and ℓ_1_-regularized partial correlation estimation.

We also apply the GRM to learn the brain A*β* network of AD patients and cognitively normal elderly subjects. The features of structural connectivity derived from PiB-PET data are particularly promising for the detection of early stage AD [6]. Our analysis on 30 AD and 40 normal control (NC) subjects supports the effectiveness of our learning method. First, we compare the classification performance of the GRM with that of other approaches. The GRM achieves the best performance among those methods. Then, we examine hub distributions of the thresholded networks. It turns out that GRM produces more balanced network structures, in contrast to those from sample correlation matrices. Moreover, the hubs of the reconstructed networks obtained by the GRM match the cortical hubs given by functional MRI. In addition, we compare the connectivity discrepancies between the networks of the AD and NC groups. The consistent differentiable network features discovered through the GRM might provide biomarkers that could classify normal, preclinical and clinical subjects more distinctively. The presented work is a significant extension of [23], where we only learned the A*β* network of AD patients and compared the algorithm with the sample correlation approach.

## Methods

We first define a notion of signals supported on graphs before presenting the proposed GRM. The relationship between the regression model and several existing methods is also discussed. Afterwards, we present the computational method used for estimating the optimal graph.

### Signals and Fourier transform on graphs

Traditional signal processing focuses on signals that are defined in Eucliden spaces. The Fourier transform, which decomposes a function into a series of harmonic sinusoids, plays a critical role in this area. Although they have achieved great success, classical signal processing methods do not meet the need of processing signals with complex intrinsic structures, for example brain images, genetic data, and sensor network measurements. This leads to a trend towards signal precessing techniques on graphs [22, 24].

To represent the brain connectivity, we introduce a weighted graph and the associated matrices that describe its structure. Let 𝓥 (|𝓥| = *N*) be a set of brain regions and ℰ be a set of edges expressing their associations. We can characterize the structure of brain imaging data by a *weighted* graph 𝓖(𝓥, ℰ, ***W***), where the weighted adjacency matrix ***W*** with *W*_*ij*_ ≥ 0 quantifies the similarity between vertices *i* and *j* (*W*_*ii*_ = 0, for all *i*). We assume that the similarity is symmetric, meaning that *W*_*ij*_ = *W*_*ji*_ for every pair of *i*, *j*. Thus, 𝓖 is an undirected graph. In addition, we denote by ***D*** the degree matrix, which is diagonal with *D*_*ii*_ = ∑_*j*_ *W*_*ij*_ corresponding to the degree of vertex *i*. Based on this, the *graph Laplacian* matrix ***L*** is defined by $L\overset{\text{def}}{=}D-W$. In spectral graph theory, numerous combinatorial and topological properties of graphs are connected to the eigenvalues and eigenvectors of the graph Laplacian [25, 26]. Moreover, the Laplacian matrix can be viewed as a *differential operator* on graph, serving as a counterpart of the classical Laplace differential operator Δ in Euclidean spaces. In mathematical physics, the heat equation $\frac{\partial u}{\partial t}=-\Delta u$ describes the distribution of heat (or many other diffusion processes) in a given region over time, with *u* being a function of spatial coordinates and time *t*. The Laplacian matrix ***L*** of a graph associated with the domain of the diffusion can be obtained by discretizing the heat equation [27].

When the graph 𝓖 is a ring graph (namely, ***W*** is a circulant matrix with the first row being [0, 1, 0, ⋯, 0, 1]), an interesting observation is that the eigenvectors of its Laplacian matrix are exactly the bases of the discrete Fourier transform (DFT) [26]. The classical DFT is the expansion of a length-*N* sequence *x*(0), ⋯, *x*(*N*−1), in terms of the Fourier basis vectors, *i.e.*, ${\hat{x}}_{\text{DFT}}(k)=\sum_{n=0}^{N-1}x(n)e^{-2\pi i\frac{k}{N}n}$. Analogously, we can introduce signals supported on graphs and the associated *graph Fourier transform* (GFT) as follows. Let ℋ(𝓥) be a Hilbert space defined on 𝓥. A signal ***x*** ∈ ℋ(𝓥) is a *N* × 1 vector, where each entry is a real value *x*_*i*_ assigned to vertex *i*. Since the Laplacian matrix is symmetric, we can diagonalize it into ***L*** = ***F*** **Λ** ***F*** ^*T*^, where **Λ** is a diagonal matrix with Λ_*ii*_ being the *i*th smallest eigenvalue of ***L***, and ***F*** is a matrix whose columns are the corresponding eigenvectors. Furthermore, since ***L*** is a positive semi-definite matrix [25], its eigenvalues are nonnegative. The GFT of ***x*** takes the form of $\hat{x}=F^{T}x$, *i.e.*, a projection of the signal to the space spanned by the eigenvectors of the graph Laplacian. In neuroimaging study, it can also be viewed as a decomposition of a brain imaging data into different modes on the brain connectivity network. Accordingly, we have the inverse GFT: $x=F\hat{x}$ [22, 24].

### Signal variation metric

In the classical Fourier transform, a smooth signal in the time domain exhibits a relatively fast decay of the magnitudes of Fourier coefficients in the frequency domain. We extend this notion of signal smoothness to signals defined on graphs via spectral graph theory.

We denote by *λ*_*i*_ and ***f***_*i*_ the *i*th eigenvalue and eigenvector of ***L***. For connected graph, we have 0 = *λ*_1_ ≤ ⋯ ≤ *λ*_*N*_ [25]. A key property that will be further justified is that the variation of ***f***_*i*_ gets larger as *i* increases, when we perceive the signal variation on graphs from the differences between values of each vertex and its neighboring vertices. Consequently, we also refer to the eigenvectors ***f***_*i*_s as frequency components of the GFT. Moreover, we define the bandwidth of a signal ***x*** as the maximum eigenvalue *λ*_*i*_ such that $f_{i}^{T}x\neq 0$. A *low-pass* signal whose GFT has an energy concentration on low frequency components is *smooth* on 𝓖 [22, 24]. To quantify the signal variation, we propose the following metric

$$\begin{array}{c} {𝓜}_{𝓖}\left(x\right)=\frac{x^{T}L^{s}x}{x^{T}x}, \end{array}$$ (1)

where *s* > 0 is an adjustable parameter. Since $x^{T}L^{s}x={\hat{x}}^{T}{\Lambda \;}^{s}\hat{x}=\sum_{i}\lambda_{i}^{s}{\hat{x}}_{i}^{2}$, we have $\|f_{i}{\|}_{𝓖}=\lambda_{i}^{s}$. The above facts indicate that indeed a higher frequency component has a larger variation on the associated graph. Hence, shrinking the variation of a signal means suppressing its high frequency components. Moreover, *s* controls the impact of eigenvalues of ***L***. In a smooth signal regularization, if we increase *s*, there will be a greater penalty on higher frequency components of ***x***.

From another perspective, a small variation in Eq (1) reflects a better fit between the graph structure and the signal, assuming that the signal is low-pass on the graph. To further interpret the variation metric and build connection to other techniques, we examine the cases when *s* = 1, 2. By the definition of graph Laplacian, we obtain (1) ***x*** ^*T*^ ***L*** ***x*** = ∑_*i* ≤ *j*_ *W*_*ij*_(*x*_*i*_−*x*_*j*_)^2^; (2) $x^{T}L^{2}x=\|(D-W)x{\|}_{2}^{2}=\sum_{i}{\left(D_{ii}x_{i}-\sum_{j}W_{ij}x_{j}\right)}^{2}$. In other words, fitting the signal to a graph will enforce an equalization (*s* = 1) or a linear approximation (*s* = 2) between values of neighboring vertices, respectively. Furthermore, when *s* = 1, the numerator in Eq (1) becomes the regularization term of Tikhonov regularization [21, 28]; when *s* = 2, the objective function of locally linear embedding (LLE) turns into a special case of the above numerator [29].

**Fig 1:**
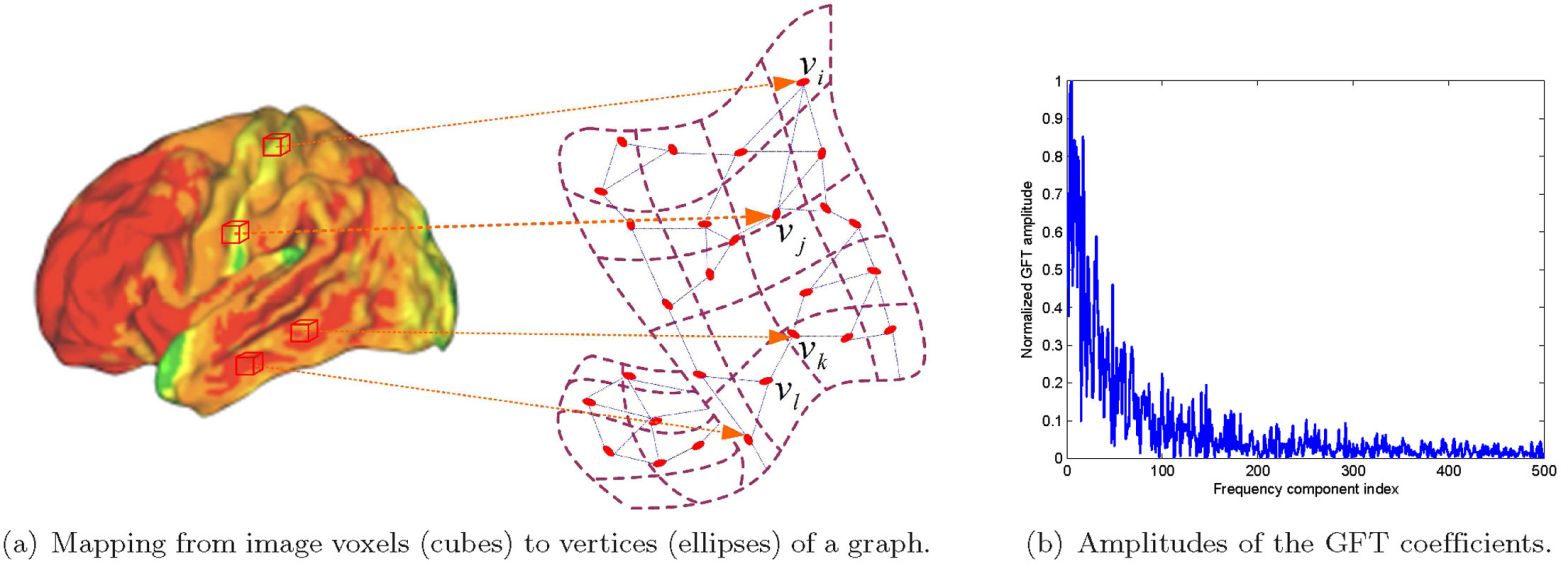
An example of smooth signal on graph. The left panel illustrates the mapping a 3D-PET image to a weighted graph that describes the affinity between every pair of voxels, exemplified by the 3D brain image in [6]. The amplitudes of the first few GFT coefficients of the image are displayed in the right panel, where the amplitudes have been divided by the the maximum value among them.

The assumption that image data are in proximity to a submanifold, and thus are smooth signals on a weighted graph in a discretized approximation, has been verified in many relevant works [27, 30, 31]. The weighted graph could be constructed by the procedures illustrated in Fig 1, exemplified by the 3D brain image in [6]. We first map each voxel of this 3D brain image to a vertex of a weighted graph as shown in Fig 1(a). The weight of the edge linking a certain pair of vertices is computed from the differences of the neighborhoods of the associated voxels, as in the graph construction based on 2D images explained in [30]. The amplitudes of the GFT coefficients of the image are displayed in Fig 1(b), where the decay of the amplitudes demonstrates the smoothness assumption.

In addition, we can justify the smoothness of the imaging data on brain connectivity networks as follows. Through analysis of large amount of data, it has been demonstrated that the propagation of disease agents of AD obeys a network diffusion model [32, 33]. By using linear dynamics defined over the brain network, Raj *et al.* predicted spatially distinct “persistent modes” of different types of dementia accurately. Meanwhile, it is also shown that the smoothness of the signal on graph will increase as the diffusion process continues [24]. Mathematically, the dynamics of many neuroimaging data such as amyloid-*β* deposition measured by PiB-PET or brain atrophy measured by MRI can be modeled by a diffusion process on graph, as governed by the following differential equation

$$\begin{array}{c} \frac{dx}{dt}=-Lx, \end{array}$$ (2)

where ***L*** is the graph Laplacian defined before. With the initial condition ***x***_init_ = ***δ***_*u*_0__ representing a unit input at vertex *u*_0_, the solution to Eq (2) is

$$\begin{array}{c} x_{t}=e^{-tL}\delta_{u_{0}}=\sum_{i=1}^{N}e^{-t\lambda_{i}}f_{i}f_{i}^{T}\delta_{u_{0}}, \end{array}$$ (3)

where again ${\left\{\lambda_{i}\right\}}_{i=1}^{N}$ are the eigenvalues of ***L*** and ${\left\{f_{i}\right\}}_{i=1}^{N}$ are the corresponding eigenvectors. From Eq (3), as time *t* increases the contribution of the higher frequencies, namely ***f***_*i*_s associated with relatively larger *λ*_*i*_s, will decrease quickly. This makes the observed signals at time *t* smooth on the graph.

### Graph regression model

In many applications, the graph structure for the observed signals is unknown. The inverse problem of learning the graph from data (*a.k.a., graph regression*) is a fundamental task that helps discover the relationship among physical units (*e.g.*, brain regions) that yield the data. We introduce a GRM with regularization on the uniformity of edge weights in this section.

We present the GRM by considering a brain connectivity network. Let {*V*_1_, ⋯, *V*_*N*_} be the *N* brain volumes-of-interest (VOIs) in our study and assume we have *M* samples. The observation taken from the *n*th region of subject *m* is denoted by ${\tilde{X}}_{n,m}$, which is an entry of the data matrix ${\tilde{X}}_{N\times M}$. We assume that ***X*** is obtained by normalizing the Euclidean norm of every column of $\tilde{X}$ to 1. Thus, it follows that $\sum_{m=1}^{M}{ℳ}_{𝓖}(x_{m})=\text{tr}(X^{T}L^{s}X)$, where $x_{m}={\left(X_{1,m},\cdots ,X_{N,m}\right)}^{T}$ is the measurement of subject *m*. Then, we formulate the GRM as

$$\begin{array}{ccc} & & \min_{L}\;\;\text{tr}\left(X^{T}L^{s}X\right)-\beta {∥W∥}_{F}^{2}, \end{array}$$ (4)

$$\begin{array}{ccc} & & \text{s.t.}\;\;\;\;\text{tr}(L)=N,\;\;L\cdot 1=0, \end{array}$$ (5)

$$\begin{array}{c} \;\;\;\;\;\;\;\;L_{ij}=L_{ji}\leq 0,\;\forall i\neq j, \end{array}$$ (6)

where ‖⋅‖_*F*_ denotes the Frobenius norm. The first term in the objective function fits graph 𝓖 to the data by minimizing the *total variation* of observed signals on 𝓖, while the second term controls the uniformity of the connection weights. To prevent the solution of a null graph, we normalize the sum of all the connection weights, *i.e.*, the trace of ***L*** in Eq (5). If *β* > 0, it follows that we tend to amplify $\|W{\|}_{F}^{2}$, making *W*_*ij*_s more nonuniform. This leads to a shrinkage of most weights, leaving a few prominent ones. An opposite effect is achieved by a negative *β*. Analogously to the Tikhonov regularization or ridge regression [28, 34], we make the above optimization problem more stable by regularizing the Frobenius norm of ***W***. The parameter *s* governs the decay speed of the GFT amplitudes of the observed signals on the estimated graph. A larger *s* will enforce a faster decay, whereas it may reduce the stability of the solutions similar to the situation we face when fitting a high-order polynomial to a limited number of data points. Thus, normally we choose 1 ≤ *s* ≤ 3.

The original learning problem presents a challenge to our analysis, since the derivative of the regularization term in Eq (4) with respect to ***L*** does not have a simple closed-form. To get around this, we slightly change the objective function to

$$\begin{array}{c} T\left(L\right)=\text{tr}\left(X^{T}L^{s}X\right)-\beta {∥L∥}_{F}^{2}, \end{array}$$ (7)

where we regularize the sum of squares of all the entries in the graph Laplacian. Because the sum of each row of ***L*** is zero, if we shrink or amplify the weights of edges associated with a certain vertex, its degree will vary accordingly. Hence, we are able to regularize the off-diagonal and diagonal entries of ***L*** together. For the altered objective function, the derivative is given by

$$\begin{array}{c} \frac{\partial T}{\partial L}=\sum_{r=0}^{s-1}L^{r}\left(XX^{T}\right)L^{s-r-1}-2\beta L. \end{array}$$ (8)

Since the constraints in (5) and (6) are linear, the feasible region specified by them is convex [35]. Hence, we can apply the *projected gradient descent* method discussed in [36] to search for optimal solutions. For a continuously differentiable objective function and linear constraints, it has been proven that the projected gradient descent method forces the sequence of projected gradients to zero and the limit points of this method are stationary points [36, Theorem 2.3 and 2.4].

## Results and Discussions

In this section, we verified the GRM by synthetic data and applied it to learn the structural brain connectivity of AD patients and NC subjects.

### Results on simulated data

We first verified our GRM based on a series of simulated data sets, which consist of *M* = 10 up to *M* = 300 signals on a random weighted graph. The random graph was built through two steps. First, we assumed that there were *N* = 16 vertices in the graph and constructed the graph structure by connecting each pair of the vertices with a probability of 0.3. Then, if two vertices were connected, a random weight uniformly drawn from 0 to 1 was assigned to the associated edge. After that, the signals were generated from linear combinations of the first 6 eigenvectors of the graph Laplacian ***L***, according to

$$\begin{array}{c} x=3\sum_{i=1}^{3}\alpha_{i}f_{i}+\sum_{j=4}^{6}\alpha_{j}f_{j}, \end{array}$$ (9)

where ***f***_*i*_ is the *i*th eigenvector of ***L***, *α*_*i*_ (1 ≤ *i* ≤ 6) is a uniform random variable in [0, 1]. We placed higher weights on the first 3 eigenvectors to make the signals smoother on the graph. Note that this choice was not essential, as long as we generated smooth signals on the graph.

For comparison purposes, we considered two other popular approaches for inferring network structures besides the proposed method. The first one infers the network by computing the sample correlation in two steps. First, it calculates the sample covariance matrix according to

$$\begin{array}{c} S=\frac{1}{M-1}\sum_{m=1}^{M}(x_{m}-\bar{x}){\left(x_{m}-\bar{x}\right)}^{T}, \end{array}$$ (10)

with $\bar{x}=\sum_{m=1}^{M}x_{m}$. Then, each element of the correlation matrix ***C*** is estimated as $C_{ij}=\frac{S_{ij}}{\sqrt{S_{ii}S_{jj}}}$. The second one estimates the partial correlation with *ℓ*_1_-regularization, since the sample correlation matrix is often singular due to the small sample size compared with the data dimension. This approach is also known as graphical Lasso [13]. For convenience, we use abbreviations *SC* and *GLasso* to indicate sample correlation and graphical Lasso in some of the figures. Given the sample covariance matrix *S* and a regularization parameter *ρ*, it maximizes the penalized log-likelihood

$$\begin{array}{c} \log\det\Theta -\text{tr}\left(S\Theta \right)-{\rho ∥\Theta ∥}_{1}, \end{array}$$ (11)

over all positive semi-definite matrices **Θ**, to obtain an estimation of the inverse of the covariance matrix. The regularization parameter *ρ* in Eq (11) controls the tradeoff between the likelihood of the data and the sparsity of the network connections.

**Fig 2:**
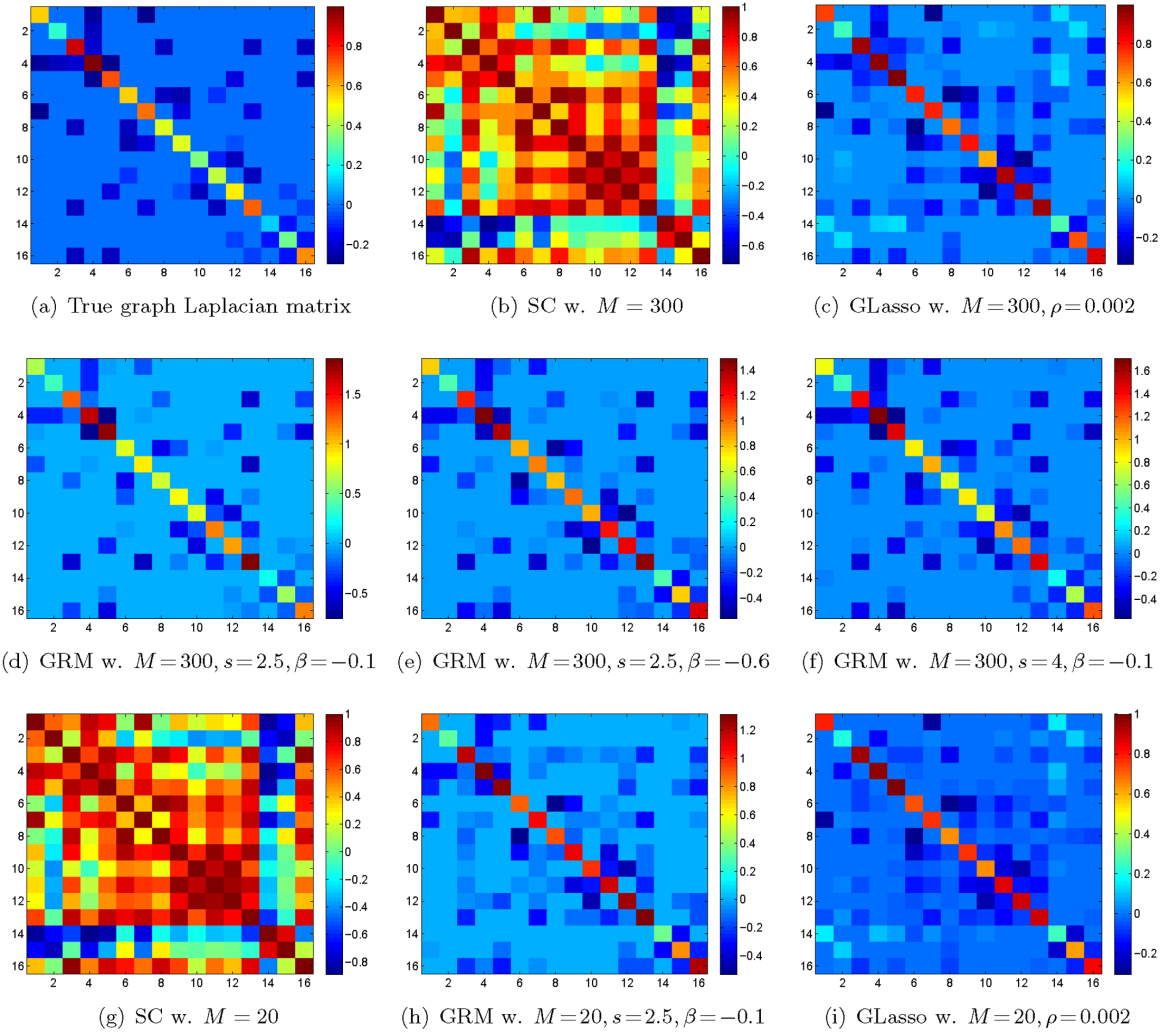
Simulation results. Simulation results on a 16-vertex random graph with its Laplacian matrix shown in (a). The data included *M* = 300 signals on the graph for (b)–(f) and *M* = 20 signals for (g)–(i). Each signal was a linear combination of the first 6 eigenvectors of the graph Laplacian. For *M* = 20 or 300, the GRM significantly outperforms the compared methods, namely the sample correlation and the graphical Lasso.

**Fig 3:**
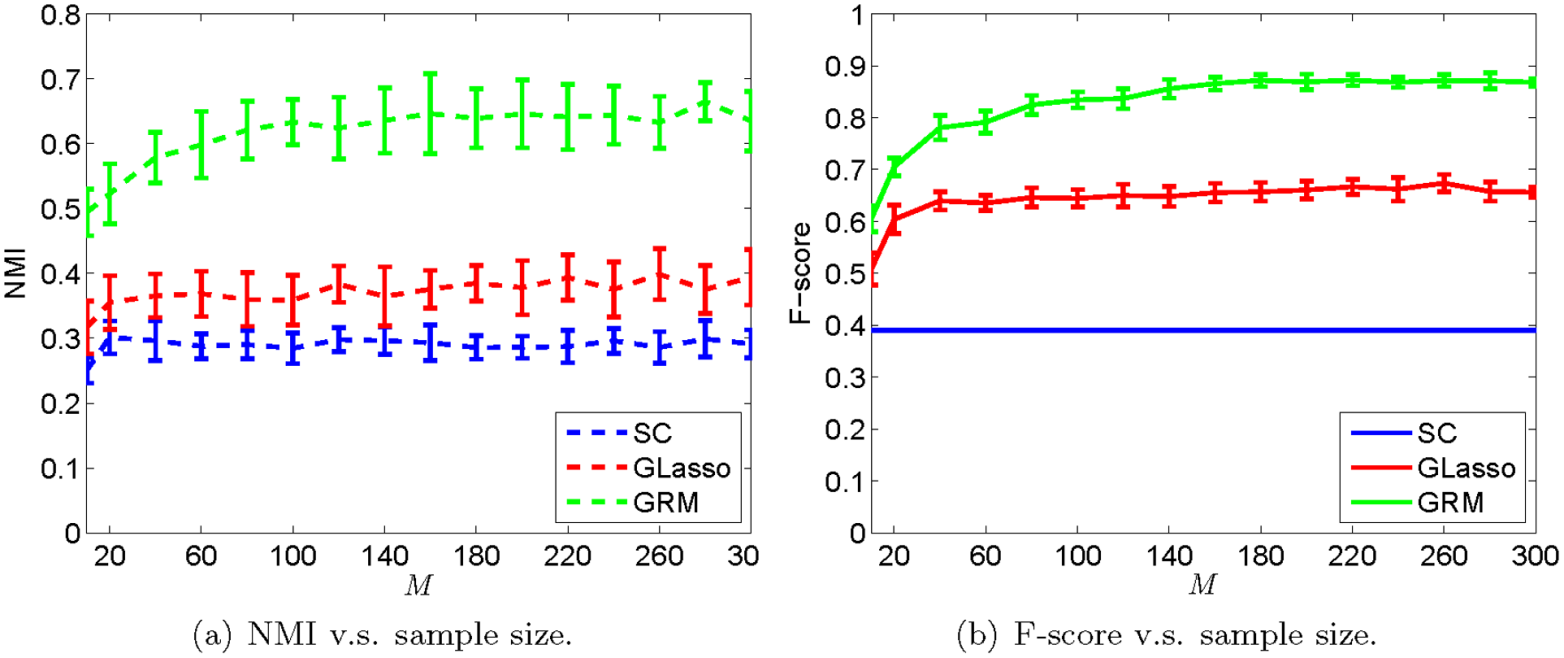
Performance of the graph structure learning methods v.s. the number of samples. In the left and right panels, we show the NMI and F-score between the estimated graph and the truth, respectively. The error bar indicates the standard deviation of the measurement. For every *M*, we ran the experiment 100 times by randomly generating the graph structure and the signals.

The simulation results of the above methods are shown in Figs 2 and 3. We tuned parameters of both GRM and graphical Lasso by maximizing the average performance of each algorithm when *M* varied from 10 to 300. When the number of samples *M* = 300, we observe that our reconstruction of ***L*** in Fig 2(d) resembles the ground truth in Fig 2(a) with *s* = 2.5, *β* = −0.1, while a simple correlation estimator in Fig 2(b) gives us visually noisy results. Fig 2(c) is the estimate of the partial correlation with *ℓ*_1_-regularization, when *ρ* = 2 × 10^−3^. In order to determine a reasonable regularization parameter in Eq (11), we repeated the learning process with different values of *ρ* and found that *ρ* = 2 × 10^−3^ yielded the best results for a wide range of *M*. The performance of the algorithm was evaluated by the Normalized Mutual Information (NMI) [37] and F-score [38] between the graph learned from the data and the ground truth. Both criteria have been commonly used in information retrieval [39]. NMI is defined according to the following formula

$$\begin{array}{c} \text{NMI}\left(A,B\right)=\frac{2\cdot \text{MI}(A,B)}{\text{H}(A)+\text{H}(B)}, \end{array}$$ (12)

where $\text{MI}(A,B)=\sum_{a\in A}\sum_{b\in B}p(a,b){\text{log}}_{2}(\frac{p(a,b)}{p(a)p(b)})$ represents the mutual information between matrix ***A*** and ***B***; H(***A***) = ∑_*a*∈***A***_ *p*(*a*)log_2_ *p*(*a*) indicates the entropy of ***A***. Here *a* and *b* are arbitrary entries in the respective matrices; *p*(⋅) denotes a probability measure (in practice, the probabilities are obtained from histogram). In addition, F-score is defined as

$$\begin{array}{c} \text{F-score}=\frac{2\cdot \text{TP}}{2\cdot \text{TP}+\text{FP}+\text{FN}}, \end{array}$$ (13)

where TP, FP and FN are the numbers of true positives, false positives, and false negatives, respectively. NMI assesses the similarity between the estimated graph and the true graph by checking the frequency that we see a certain pair of connection weights at the same corresponding locations, while F-score measures the similarity by checking if we correctly recover the locations of the edges. We used these two metrics to obtain a more comprehensive comparison between different algorithms.

In the above simulation, when *M* = 300, we see that the NMI and F-score of Fig 2(d) are (0.6973, 0.8824). Meanwhile, these two measurements of Fig 2(e) and 2(f) are (0.6097, 0.7895) and (0.6040, 0.7895), respectively. By searching different combinations of *s* and *β*, we chose *s* = 2.5, *β* = −0.1 for the GRM. When the number of samples is very small, namely *M* = 20, the NMI and F-score for the GRM are (0.4668, 0.6667) in Fig 2(h). In contrast, those two measurements for the sample correlation in Fig 2(g) and the graphical Lasso in Fig 2(i) are (0.3160, 0.3899) and (0.3161, 0.5806), accordingly. Furthermore, we compared the NMI and F-score by varying the sampling size *M* from 10 to 300 with a step-size 10. For each *M*, we ran the experiment multiple times to take the randomness of the graph realization and data generation into account. The results are presented in Fig 3, where the left and right panels illustrate the trend of the NMI and the F-score, respectively. We can see that the GRM outperforms the sample correlation and graphical Lasso significantly, even when *M* = 10. This verifies that our proposed approach can learn a more consistent graph under insufficient samples.

### AD and NC study

Table 1 provides the demographics of the cognitively normal elderly control subjects and the participants with Alzheimer’s disease. All participants of this study were recruited from ongoing neuroimaging studies in aging and during screening for dementia clinical trials at the Massachusetts General and Brigham and Women’s Hospitals [6]. The evaluation of the participants included a ^11^C-PiB-PET scan. All subjects were provided written informed consent, prior to any experimental procedures, in accordance with protocols approved by the Institutional Review Board of Partners Healthcare Inc. The study was approved by and conducted under the auspices of the Partners Human Research Committee at Brigham and Women’s Hospital and Massachusetts General Hospital. Each cognitively normal elderly subject was required to have a normal neurological examination, a Clinical Dementia Rating (CDR) [40] scale score of 0, a Mini-Mental State Examination (MMSE) over 27 and performance within 1.5 SD on age and education-adjusted norms on cognitive testing [41]. In addition, there was not any notable medical or neuropsychiatric illness, nor was there a history of alcoholism, drug abuse, head trauma, or a family history of autosomal dominant Alzheimer’s disease found from the cognitively normal elderly participants. Each individual with Alzheimer’s disease showed a CDR scale of 1 and met criteria for a clinical diagnosis of probable Alzheimer’s disease according to the National Institute of Neurological and Communicative Disorders and Stroke/Alzheimer’s Disease and Related Disorders Association criteria [42].

**Table 1:** Participant demographics.

	Sample size	Avg. age (SD)	Avg. education (SD)	Avg. MMSE (SD)	Avg. CDR
AD	30 (13 male)	73.03 (7.99)	17.5 (1)	25 (1.63)	1
NC	40 (14 male)	76.15 (8.04)	16.24 (2.88)	29.05 (1.1)	0

AD = Alzhemier’s Disease; NC = Normal Control subjects; SD = Standard Deviation; MMSE = Mini-Mental State Examination; CDR = Clinical Dementia Rating. The age and education are measured in years.

All acquisition, preprocessing and image analysis of ^11^C-PiB-PET were conducted using standard procedures as published previously (see below for detailed references). The PET scans were acquired on a Siemens/CTI ECAT HR^+^ PET scanner at Massachusetts General Hospital [6]. Amyloid imaging was performed with N-methyl-^11^C-2-(4-methylaminophenyl)-6-hydroxybenzothiazole (PiB). ^11^C-PiB was prepared as described by [43]. Briefly, after a transmission scan, 10–15mCi ^11^C-PiB was injected as a bolus intravenously, which was followed immediately by a 60-min dynamic PET scan in 3D mode (63 image planes, 15.2cm axial field of view, 5.6mm transaxial resolution and 2.4mm slice interval; 69 frames: 12 × 15s, 57 × 60s). PiB-PET data were reconstructed with ordered set expectation maximization, corrected for attenuation, and each frame was evaluated to verify adequate count statistics and absence of head motion. Individual ^11^C-PiB-PET scans were spatially normalized using the warping information derived from individual stereotactic normalization of the structural MRI using SPM 5.

As shown in Table 1, the data set consists of 30 AD patients and 40 NC subjects. PET neuroimaging data were downsampled from the normalized 2 mm isotropic voxels to 8 mm isotropic voxels for computational efficiency. Each image has a dimension of 20 × 24 × 18 with 8mm × 8mm × 8mm voxels. In the data preprocessing step, we first masked out the area outside of the brain. Next, we applied Automated Anatomical Labeling (AAL) [44] to map the effective voxels to 116 VOIs. The data were then averaged within each VOI for further analysis. Among all the VOIs, we picked up 42 regions that were considered to be potentially related to AD [45]. Those regions are distributed in the frontal, parietal, occipital, and temporal lobes. Table 2 lists the names of the selected VOIs and their corresponding lobes. The numbers of selected VOIs in the above four brain lobes are 12, 8, 6, and 16, respectively.

**Table 2:** Names of the VOIs used in the brain connectivity learning model.

	**Frontal Lobe**		**Parietal Lobe**		**Occipital Lobe**		**Temporal Lobe**
1	L Superior Frontal Gyrus	13	L Superior Parietal Lobule	21	L Superior Occipital	27	L Superior Temporal Gyrus
2	R Superior Frontal Gyrus	14	R Superior Parietal Lobule	22	R Superior Occipital	28	R Superior Temporal Gyrus
3	L Middle Frontal Gyrus	15	L Inferior Parietal Lobule	23	L Middle Occipital Gyrus	29	L Superior Temporal Pole
4	R Middle Frontal Gyrus	16	R Inferior Parietal Lobule	24	R Middle Occipital Gyrus	30	R Superior Temporal Pole
5	L Middle Frontal Gyrus	17	L Precuneus	25	L Inferior Occipital Cortex	31	L Middle Temporal Gyrus
6	R Middle Frontal Gyrus	18	R Precuneus	26	R Inferior Occipital Cortex	32	R Middle Temporal Gyrus
7	L Middle Orbitofrontal Gyrus	19	L Posterior Cingulate Gyrus			33	L Middle Temporal Pole
8	R Middle Orbitofrontal Gyrus	20	R Posterior Cingulate Gyrus			34	R Middle Temporal Pole
9	L Gyrus Rectus					35	L Inferior Temporal Gyrus
10	R Gyrus Rectus					36	R Inferior Temporal Gyrus
11	L Anterior Cingulate Gyrus					37	L Fusiform Gyrus
12	R Anterior Cingulate Gyrus					38	R Fusiform Gyrus
						39	L Hippocampus
						40	R Hippocampus
						41	L Parahippocampal Gyrus
						42	R Parahippocampal Gyrus

L = Left hemisphere; R = Right hemisphere.

### Brain connectivity learning by GRM

**Fig 4:**
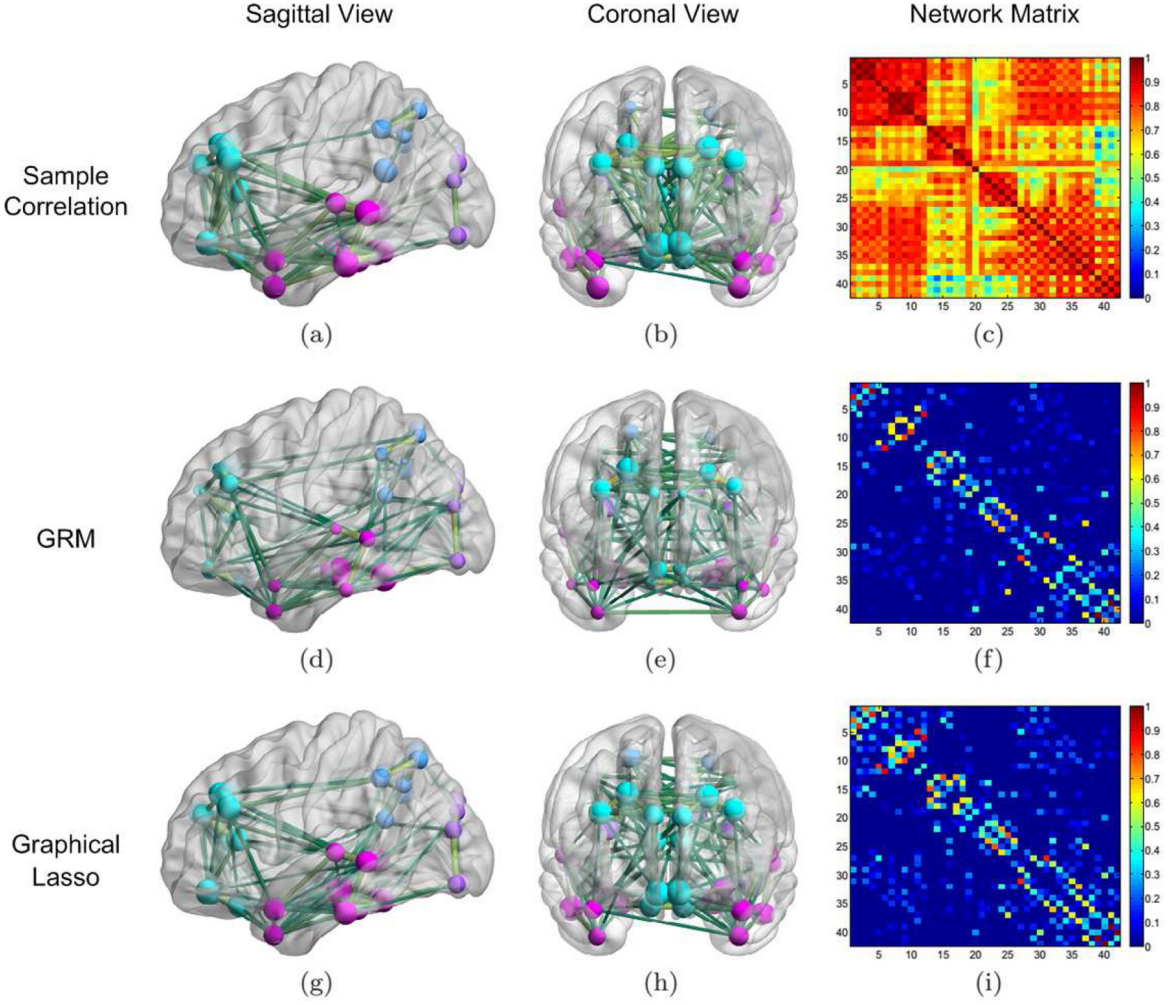
Network learning results of the AD group. Reconstruction results by the GRM (2nd row) from 30 AD subjects compared with those by sample correlation (1st row) and graphical Lasso (3rd row). We plotted the sagittal and coronal views of the thresholded networks in the first two columns, where 144 prominent links are shown for all cases. The colors and sizes of the nodes indicate the associated brain lobes (frontal lobe is cyan; temporal lobe is pink; parietal lobe is blue; occipital lobe is purple) and node degrees, respectively; the thicknesses of the edges encode the connection strengthes. Those figures were generated by BrainNet Viewer (http://www.nitrc.org/projects/bnv/). We used *s* = 2, *β* = −0.003 and *ρ* = 0.002 to simulate (f) and (i), respectively. To ease visualization, we removed the diagonal entries when displaying the graph Laplacian and partial correlation matrices.

Before applying the GRM to learn the brain connectivity of the AD group, we computed a correlation matrix based upon the measurements taken from the 42 VOIs of each AD patient. We show the result in Fig 4(c), where the numbering of the VOI areas was based on lobes, and the four brain lobes are distinguishable in the blocks next to the diagonal. But it is hard to set a simple threshold to obtain a meaningful binary graph, due to the inhomogeneity of the contrast of the correlation coefficients within different lobes. To better visualize the network structure, we show the strongest edges for sample correlation and the other two methods (Fig 4; columns 1 and 2).

Next, we exploited the GRM to learn the A*β* network of the AD group. We tuned the parameters of the GRM by minimizing the error of classification between AD and NC with leave-one-out cross-validation. The same procedure was carried out for determining the parameters of the graphical Lasso. When *s* = 2, *β* = −0.003, our method yielded a Laplacian matrix showed in Fig 4(f) and connectivity diagrams displayed in Fig 4(d) and 4(e) after thresholding. The choice of *β* = −0.003 in our learning model slightly spreads out the significant edges of the estimated graph; meanwhile, the choice of *s* = 2 allows us to obtain a relatively fast decay of the GFT coefficients of the data, as well as keep the solution stable (larger *s* reduces the stability).

In contrast to the noisy sample correlation, the resulting Laplacian matrix in Fig 4(f) extracts cleaner and potentially more meaningful information from the data. The absolute value of each off-diagonal term describes the associativity between each pair of brain regions. It seems that our algorithm automatically adapts a threshold to the local correlation levels and thus returns the most significant connections within each lobe. If we were to apply a simple uniform threshold to the sample correlation matrix, almost all edges within the frontal lobe and very few within the temporal lobe would remain, which is not quite reasonable. Other than keeping the prominent edges, the GRM also selects a small amount of weak edges in the correlation matrix, such as the connection between Right Posterior Cingulate Gyrus and Right Hippocampus.

For comparison purposes, we also estimated the partial correlation by using the graphical Lasso. The result with a regularization parameter *ρ* = 0.002 is displayed in Fig 4(i). We observe that while there is overlap between Fig 4(i) and 4(f) in terms of notable connections, denser connections within the frontal lobe as well as between the frontal lobe and the temporal lobe are found in Fig 4(i). In addition, it appears that the partial correlation in Fig 4(i) bears a similar structure to the correlation matrix in Fig 4(c) after thresholding.

**Fig 5:**
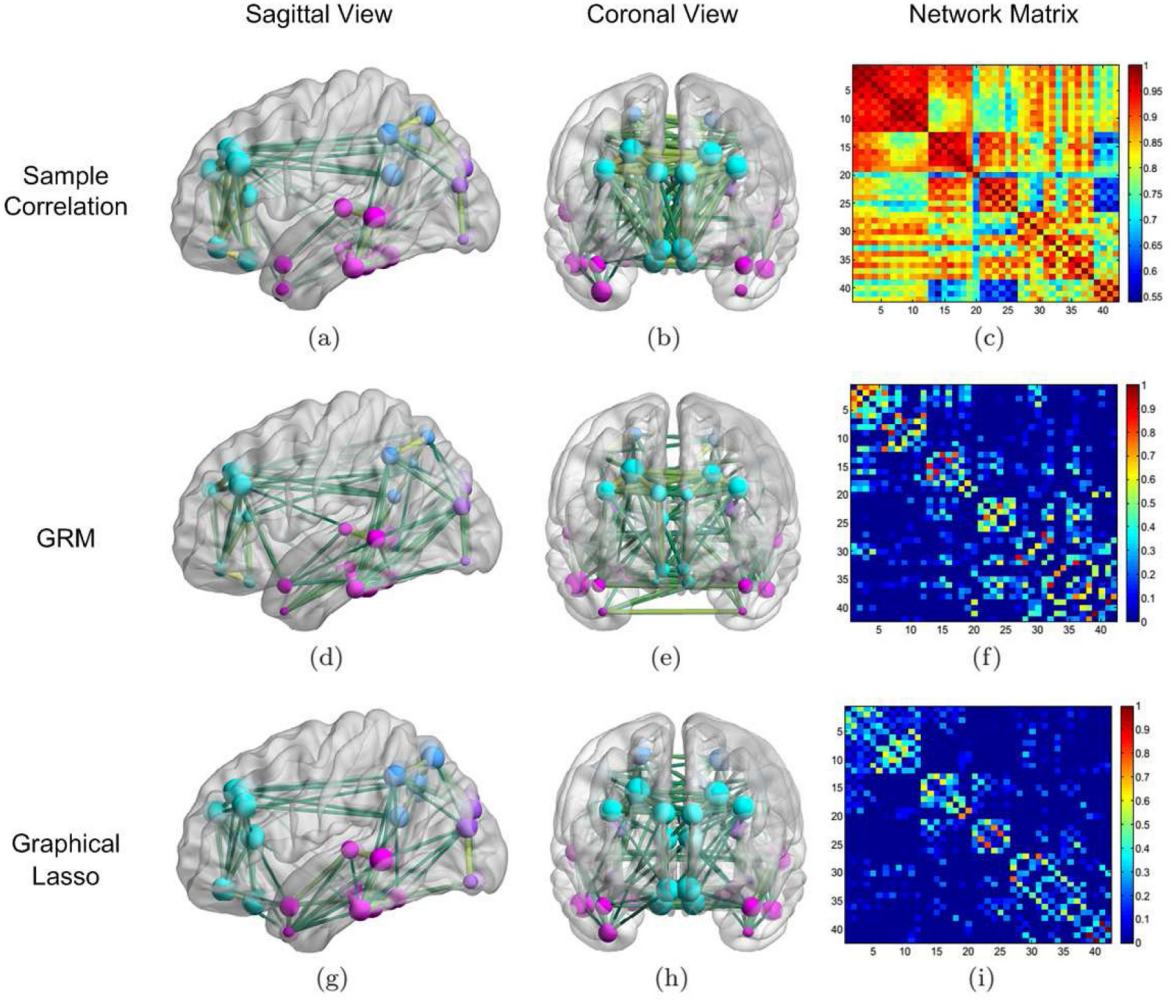
Network learning results of the NC group. Reconstruction results by the GRM from 40 NC subjects compared with those by sample correlation and graphical Lasso. For both GRM and graphical Lasso, results are obtained by keeping the same parameters as in the previous AD case. More detailed descriptions of the figure generation can be found in the caption of Fig 4.

Following the same routine, for the NC group we also computed the sample correlation and compared it with the graph Laplacian and the partial correlation given by the GRM and the graphical Lasso, respectively. The associated matrices and connectivity patterns are presented in Fig 5. Again the adjacency matrices obtained by both our method and the graphical Lasso have high contrast between the strength of the connections along diagonal and that of the off-diagonal connections. Nevertheless, in the network estimated by the thresholded sample correlation matrix (see Fig 5(a) and 5(b)), the significant connections between the occipital lobe and the temporal lobe are lost due to the thresholding. Comparing the result from the graphical Lasso in Fig 5(i) to the connections in Fig 5(f), we find that in the former network there are more connections within the frontal lobe and less connections between the occipital and the temporal lobe. Notice that in Figs 4 and 5, for getting a better contrast, we dropped off diagonal elements when displaying graph Laplacian and partial correlation matrices. Besides, only absolute edge weights are shown after a linear normalization of the maximum absolute value to one.

The networks learned by the GRM and graphical Lasso differ from each other significantly. Since many weak connections might be due to noise, we thresholded the networks learned by GRM and graphical Lasso so that both of them remained 187 edges. We will discuss the choice of thresholds when we analyze the hub locations. After thresholding, we find that the two methods merely share 57.81% (137 out of 237) of edges in the AD group and 43.85% (114 out of 260) of edges in the NC group, which means that the two methods have yielded slightly more similar network structures in the AD group when compared with the NC group. In the next subsection, we compare the GRM and the graphical Lasso in more detail.

### AD and NC classification study

**Fig 6:**
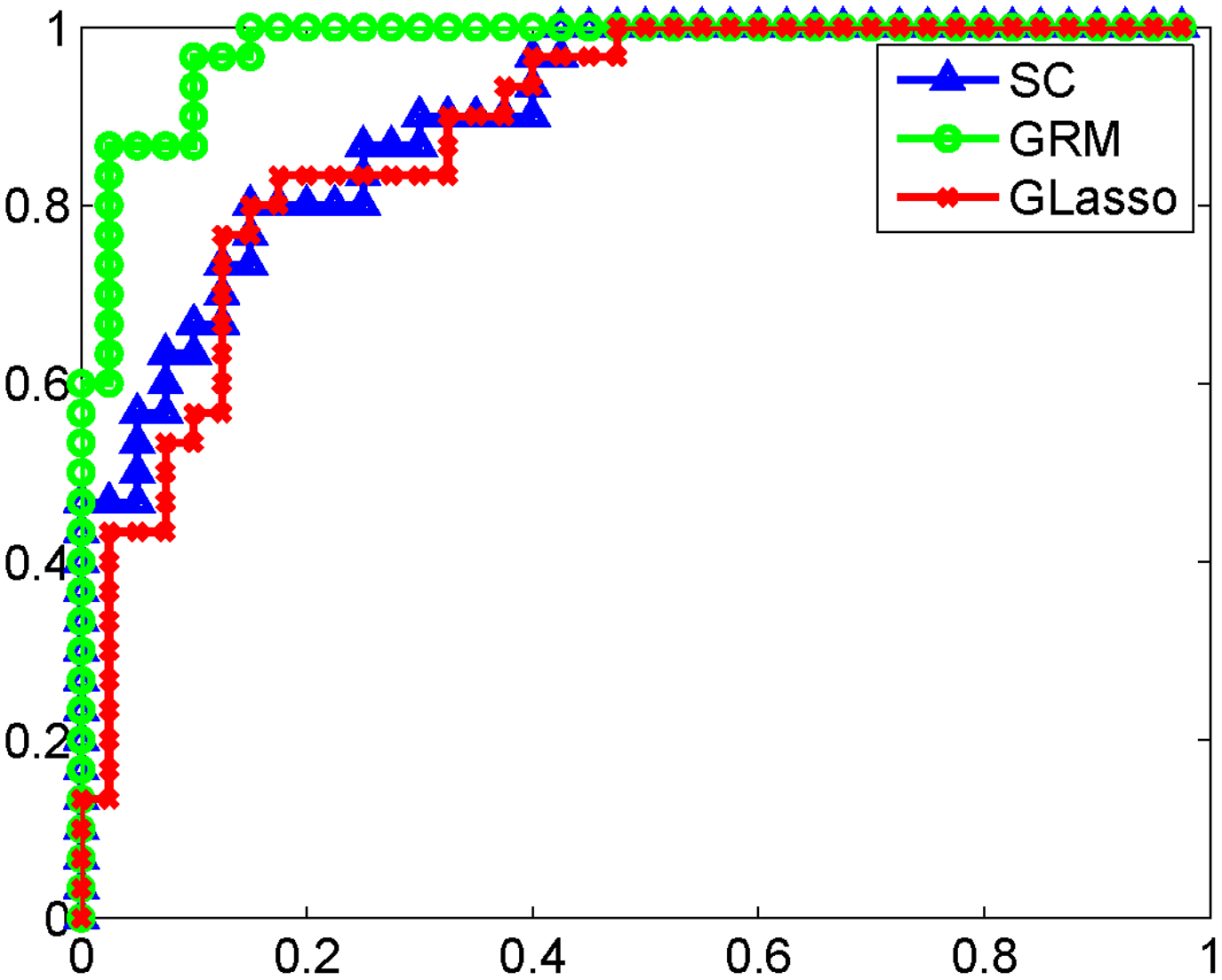
ROC curves of PiB-PET data classification. ROC curves of PiB-PET data classification for the proposed GRM and sample correlation (SC), graphical Lasso (GLasso). When using GRM, we compare the variations of the testing data point on the graphs learned from the training data of the AD group and NC group, respectively. When using SC or GLasso, we compare the Mahalanobis distances or the likelihood of the new data point under the two Gaussian graphical models, accordingly. The GRM significantly outperforms the other two methods.

To justify that our method provides effective bio-markers, we preformed a classification study of AD versus NC based on the PiB-PET data and leave-one-out tests. Each time we removed one subject from the whole data set and treated the rest of the data and the removed subject as training and testing data respectively. For the GRM, we first learned the weighted graphs of the AD group and NC group with the training data. Then, we compared the variations of a testing data point on these graphs according to (1). We classified the testing data to AD or NC group by selecting the graph that yielded a lower signal variation. The receiver operating characteristic (ROC) curve of the GRM is shown in Fig 6. We also compared its performance with that of the sample correlation and graphical Lasso. For the graphical Lasso, we computed the likelihood of a testing data point *x*_test_ under each of the learned Gaussian graphical models and chose the model that had larger likelihood. The likelihood function is defined in terms of the probability density function $f(x|\hat{\Theta \;})$ as follows:

$$\begin{array}{c} \ell \left(\hat{\Theta }|x_{\text{test}}\right)=f\left(x|\hat{\Theta }\right)={\left(2\pi \right)}^{-\frac{N}{2}}{\det(\hat{\Theta })}^{\frac{1}{2}}e^{-\frac{1}{2}{\left(x_{\text{test}}-\hat{\mu }\right)}^{T}\hat{\Theta }\left(x_{\text{test}}-\hat{\mu }\right)}, \end{array}$$ (14)

where $\hat{\Theta \;}$ is an estimate of the inverse covariance matrix; $\hat{\mu }$ is the sample mean; *N* denotes the dimension of the model. However, for the sample correlation method, the reciprocal of determinant of the sample covariance matrix is infinity, since the matrix is singular. Thus, we have calculated the Mahalanobis distances of ***x***_test_ under the two Gaussian graphical models defined below:

$$\begin{array}{c} d_{M}\left(x_{\text{test}}\right)=\sqrt{{\left(x_{\text{test}}-\hat{\mu }\right)}^{T}{\left(S+\sigma I\right)}^{-1}\left(x_{\text{test}}-\hat{\mu }\right)}, \end{array}$$ (15)

where ***S*** is the sample covariance matrix of the training data and *σ* is a small quantity to eliminate the singularity of ***S***. We tuned the parameters of the above methods optimally using grid search. The parameters that we have used in classification are listed in Table 3. The ROC curves in Fig 6 shows that the GRM can achieve a better classification result than that of the sample correlation and graphical Lasso. The latter two methods have a very close performance. We have summarized the performance metrics of those classification methods in Table 3, from which we find that our method has a significantly higher area under the curve (AUC) than that of the sample correlation or graphical Lasso, with a p-value of 0.028 or 0.014, accordingly. Meanwhile, it achieves the highest accuracy among the three methods.

**Table 3:** Performance metrics and parameters of PiB-PET data classification.

	AUC	ACR	SEN	SPE	Parameters
SC	0.9008	0.8286	0.8000	0.8500	*σ* = 0.001
GRM	0.9783	0.9286	0.9667	0.9000	*s* = 2, *β* = −0.003
GLasso	0.8825	0.8286	0.8333	0.8250	*ρ* = 0.002

AUC: area under the curve; ACR: accuracy; SEN: sensitivity; SPE: specificity.

### Network metrics computation

To further evaluate the differences among the discussed approaches, next we computed three typical network metrics: global efficiency, clustering coefficient, and maximized modularity.

It has been shown that human brain networks exhibit small-world network topologies, which provide a good balance between clustering effect and global connectivity [46, 47]. The classical small-world network model [48] proposed the clustering coefficient and the characteristic path length of binary networks. The characteristic path length is simply the average shortest path length between every pair of nodes. The information it carries can also be expressed in the global efficiency, which is the mean of the reciprocal of pairwise distances. For weighted networks, we could use the generalized clustering coefficient *C*_*wu*_ [49] and the global efficiency *E*_*global*_ [50] to assess the small-worldness of the network as defined in S1 Appendix. A small-world network has a large generalized clustering coefficient, like regular lattices, simultaneously possessing a high global efficiency, like random graphs. Recent findings have demonstrated that the loss of small-worldness is associated with AD [47]. Hence, we can employ *C*_*wu*_ and *E*_*global*_ as discriminative metrics between the networks of the AD and NC groups.

In addition, we evaluated the maximized modularity of the networks. Modularity is a quantity measuring the strength of division of a network into modules (*a.k.a.*, blocks, clusters or communities) [51]. For completeness, we include the definition of network modularity in S1 Appendix. The results of these three network measurements are listed in Table 4, where we also consider another network construction method based on normalized mutual information between measurements taken from different brain regions. We will further discuss this method in the end of this section.

**Table 4:** Measurements of the weighted graphs obtained by different methods.

Graph metric	SC (AD)	SC (NC)	GRM (AD)	GRM (NC)	GLasso (AD)	GLasso (NC)	NMI (AD)	NMI (NC)
global efficiency	0.7352	0.8257	0.1429	0.2143	0.1721	0.1581	0.6800	0.6832
clustering coefficient	0.7720	0.8230	0.0079	0.0429	0.0243	0.0267	0.6778	0.6816
maximized modularity	0.0107	0.0121	0.4743	0.4053	0.4762	0.4525	1.8 × 10^−16^	4.7 × 10^−17^

SC = Sample Correlation; GLasso = Graphical Lasso; NMI = Normalized Mutual Information. We use AD or NC inside the parentheses to denote the subject group.

It is necessary to point out that the clustering coefficient and global efficiency are dependent on the scales of the edge weights. For instance, if we double all the weights, by the definitions in S1 Appendix, the clustering coefficient and global efficiency will also be doubled. Hence, it is not meaningful to directly compare these two quantities across different methods. Nevertheless, for a given metric, we can compare its relative differences between the AD and NC groups over different graph learning algorithms.

For clustering coefficient, we observe that the value of the NC group is larger than that of the AD group among all the approaches. But the percentage difference of the GRM is the most significant: GRM (81.59%) > SC (12.27%) > GLasso (8.99%) > NMI (0.56%). For global efficiency, similarly we find that the measurement of the NC group is higher that that of the AD group in the SC, GRM, and MI. The only exception is the GLasso which results in a smaller global efficiency in the NC group. Among the three former methods, again the GRM has the largest percentage difference of the measurements: GRM (33.32%) > SC (10.96%) > NMI (0.47%). For the GRM, from the above comparison, we see that the resulting network of the AD group exhibits a remarkable loss of small-worldness in contrast to the NC group. The SC and NMI also have a similar trend in terms of the clustering coefficient and global efficiency but both with a less significance. However, the maximized modularity of either SC or NMI is much smaller than that of the GRM. This means that the modular structure of the network learned by SC or NMI is not as evident as that of the network from the GRM. The above findings of the alternation of the network efficiency and clustering coefficient from the GRM are coherent with the discovery in the literature [46, 47].

In what follows, we interpret the physiological meanings of the connectivity by studying the hub locations and comparing the thresholded networks between the AD and NC groups.

### Degree distribution and hub location study

We compared degree distributions of the weighted networks learned by different methods and evaluated the difference among the thresholded networks by investigating locations of network hubs, namely brain regions with rich connections to other areas [52–57]. These hubs are intriguing due to their potential role in information integration and relevance to brain diseases [5, 53]. For comparison purposes, we selected the same number of most significant edges in the networks given by different methods.

We first directly compared degree distributions of the weighted networks. In Table 5, we display the average degree of the vertices of each network. Although the scales of the average degrees are very different for the listed methods, we can assess the average degree difference between groups. Thus, in the third row, we have calculated the percentage difference between the AD and NC groups. We observe that the GRM has the largest relative difference of the average degree. In addition, among all the methods, only SC and GRM exhibit significant difference between groups. This result together with the global efficiency and clustering coefficient measurements in Table 4 indicate that the graphs learned by the GRM are more distinct with each other.

**Table 5:** Average degree of the weighted networks learned by different approaches.

Average degree	SC	GRM	GLasso	NMI
AD	29.1206	1.9744	2.6482	27.8782
NC	33.8538	4.1738	2.7325	28.0100
(NC-AD)/NC	13.98%	52.70%	3.09%	0.47%
p-value	1.9 × 10^−22^	7.6 × 10^−14^	0.0890	0.7932

The third row denotes the relative difference between the AD and NC groups.

**Fig 7:**
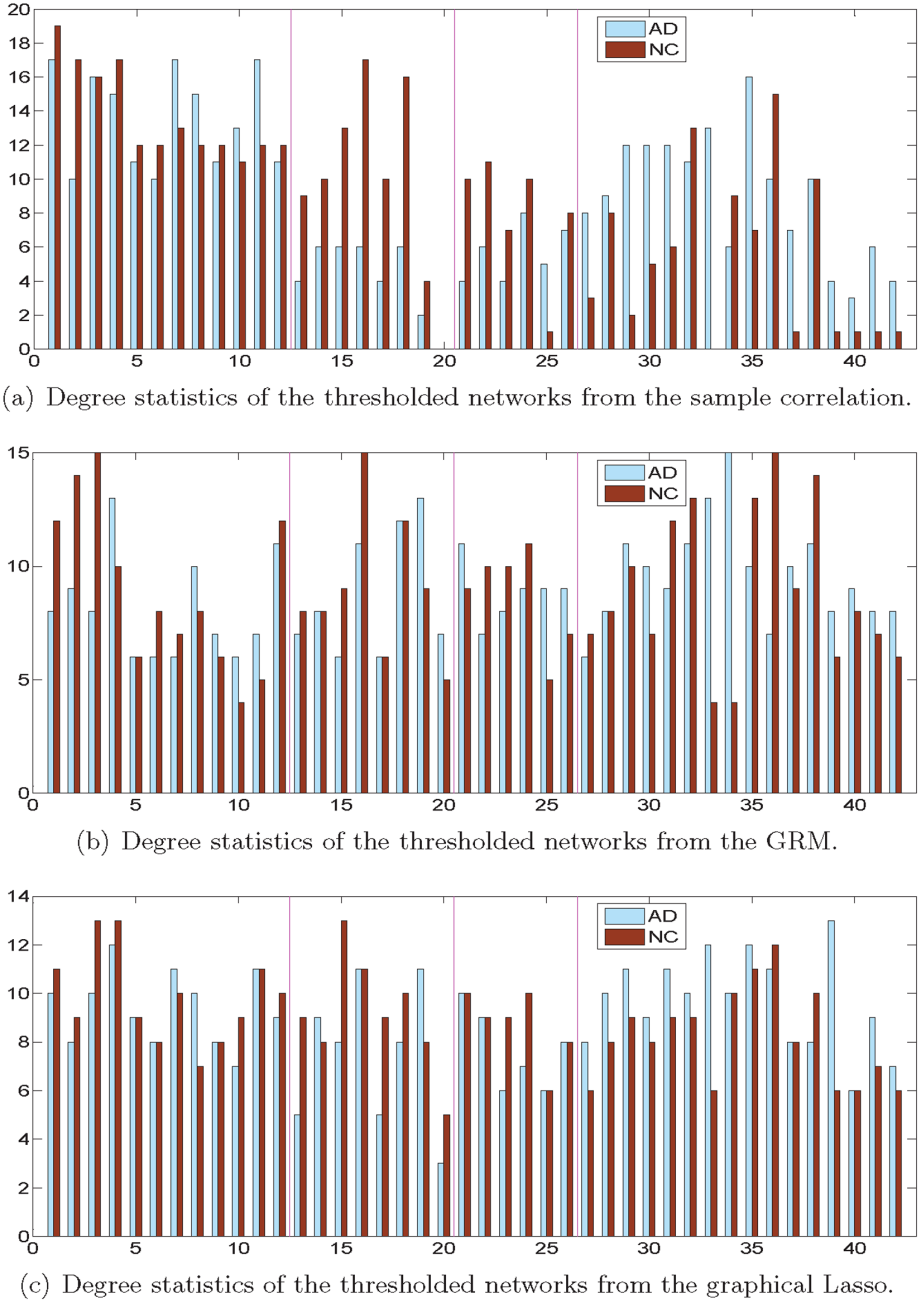
Degree distributions of the thresholded networks. Degree distributions of the thresholded AD and NC networks obtained by different methods. The horizontal and vertical axes correspond to the brain region index and vertex degree, respectively. The magenta lines delineate the four brain lobes: namely, the frontal, parietal, occipital, and temporal lobe (from left to right).

Then, we applied different thresholds to the sample correlation, the graph Laplacian and the ℓ_1_-regularized partial correlation matrices of the AD and NC groups, so that the resulting networks all had 187 edges. Here we only retained 21.7% of all possible edges in the network because we would like to focus attention on the topology of relative sparse (low-cost) networks [50], which represent the strongest functional connections [50]. This is widely justified by the known sparsity of anatomical connections in human and non-human nervous systems; for instance, in Caenorhabditis elegans, less than 6% of all possible unweighted synaptic connections exist between neurons [58]. We plotted the degree of every vertex in the associated thresholded networks of the AD and NC groups in Fig 7.

In Fig 7, by ranking the vertex degrees in the AD group network from the GRM, we discover four hubs at Right Middle Frontal Gyrus, Left Posterior Cingulate Gyrus and Left/Right Middle Temporal Pole, distributed in the frontal, parietal and temporal lobes, respectively; while the top four hubs given by the sample correlation method are Left Superior Frontal Gyrus, Left Middle Frontal Gyrus, Left Middle Orbitofrontal Gyrus, and Left Anterior Cingulate Gyrus (all belonging to the frontal lobe), which implies that the network structure is not very balanced. Besides, the hubs resulted from the graphical Lasso are Right Middle Frontal Gyrus, Left Middle Temporal Pole, Left Inferior Temporal Gyrus and Left Hippocampus, close to the hubs found by the GRM. In parallel, we compared the hubs in the NC group networks learned by different methods. From the results of the GRM, we find the top four hubs in Left Middle Frontal Gyrus, Right Inferior Parietal Lobule, Right Inferior Temporal Gyrus and Right Fusiform Gyrus; while the top four lobes discovered by the sample correlation are Left/Right Superior Frontal Gyrus, Right Middle Frontal Gyrus, Parietal Lobule. In addition, the hubs obtained by graphical Lasso are Left/Right Middle Frontal Gyrus and Left/Right Inferior Parietal Lobule. This distribution is similar to that obtained by the GRM. Nevertheless, according to the results of the AD and NC groups, the hub locations found by the GRM are more dispersed in the brain and they fit better with the distributed pathology in AD [52].

Moreover, we qualitatively compared the above outcome to the hub locations revealed by fMRI literature. Previous investigation on the fMRI correlation network disclosed that the typical cortical hubs included medial prefrontal cortex, bilateral parietal/angular cortex, posterior cingulated cortex/precuneus and bilateral lateral temporal cortex [5, 52, 57]. We observe that there is a strong correspondence between the hubs found by the fMRI and the PiB-PET data.

### Comparison between the AD and NC groups

Comparing the degree distributions of the AD and NC groups presented in Fig 7, we find that there are many nodes (mainly in the parietal, occipital, temporal lobes) with large discrepancies in degree between the two groups in Fig 7(a); nevertheless, the degree distributions of the AD and NC groups are more congruent with each other when we adopt either the GRM or the graphical Lasso to learn the network structures. According to Fig 7(b) and 7(c), some distinctive nodes between the two groups in terms of their degrees are Right Inferior Parietal Lobule and Left/Right Middle Temporal Pole (for the GRM); Left Inferior Parietal Lobule, Left Middle Temporal Pole and Left Hippocampus (for the graphical Lasso).

**Fig 8:**
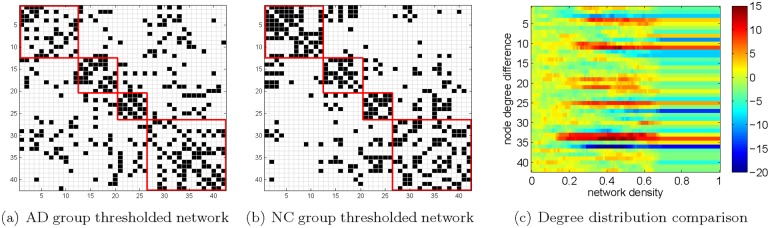
Comparison between the AD and NC networks learned by GRM. (a)-(b) correspond to the adjacency matrices of thresholded networks with both 187 edges, respectively; (c) visualizes the degree distribution differences between the AD and NC networks, with respect to the network density. Both the horizontal and vertical axes in the first two diagrams represent the brain region index.

We can also compare the brain connectivity patterns learned by GRM between the AD and NC groups in more detail. To facilitate the comparison, we solved the optimization problem Eq (7) with the constraints in Eqs (5) and (6) by setting *s* = 2, *β* = −0.003 for both groups. Then, we thresholded the resulting adjacency matrices in order to pick up the same number of significant edges from the associated networks. This ruled out the connectivity difference between the two groups due to the disparity of the scalings of the edge weights, whereas the distinction of the network organizations was preserved. Fig 8(a) and 8(b) illustrate the binary graphs after thresholding with 187 edges in both cases. Each blue cell corresponds to an edge between two vertices. Since the matrices are symmetric, the total number of blue cells is twice the number of edges in each graph. In every plot, we highlighted the four brain lobes by frames in red. From top left to bottom right, there is frontal lobe, parietal lobe, occipital lobe and temporal lobe, respectively.

In general, the resulting networks in Fig 8(a) and 8(b) both a have fewer number of intra-lobe connections than inner-lobe connections. However, there are notable discrepancies between them. First, the temporal lobe of AD has a larger amount of connections than NC. In particular, the number of connections between the temporal lobe and frontal lobe in AD group is 32, significantly larger than the number of corresponding connections 13 in the NC group. In terms of particular regions, the connections between Left/Right Middle Temporal Pole (No. 33–34) and other regions increase remarkably in the AD group. These A*β* connectivity increments can be explained by advance of the disease [6]. Although the classic regions Left/Right Hippocampus and Left/Right Parahippocampal Gyrus (No. 39–42) also have slightly denser connections in AD than NC, this trend is more prominent in Left/Right Middle Temporal Pole. Besides, we notice that the Right Inferior Temporal Gyrus (No. 36) has fewer connections to the regions outside the temporal lobe in AD than NC. We observe that the connections within the frontal lobe of AD are less than those of NC from Fig 8. In addition to the previous study, we evaluated the impact of the thresholds that we applied to the weighted networks. In Fig 8(c), we have considered the degree difference between the AD and NC groups with various network densities (*i.e.*, the number of edges after thresholding over the total number of possible edges). The horizontal axis is the percentage of edges we keep and the vertical axis corresponds to the degree of a certain brain region in the AD network minus that in the NC network. We observe that there are a few brain regions such as Anterior Cingulate Gyrus (No. 11), Right Middle Temporal Pole (No. 34), Right Inferior Temporal Gyrus (No. 36), exhibiting large discrepancies over a wide range of network densities. The above differentiable network features discovered through the GRM might provide biomarkers that can classify AD and NC subjects more distinctly.

### Other network construction

We have compared our proposed GRM with a mainstream method, namely sample correlation, and the state-of-the-art approach, namely the graphical Lasso. We notice that people also use normalized mutual information (NMI) between observed data of every two brain regions to construct brain connectivity networks. However, this might not be a good way to construct networks with a very small amount of observations, as in our case.

**Fig 9:**
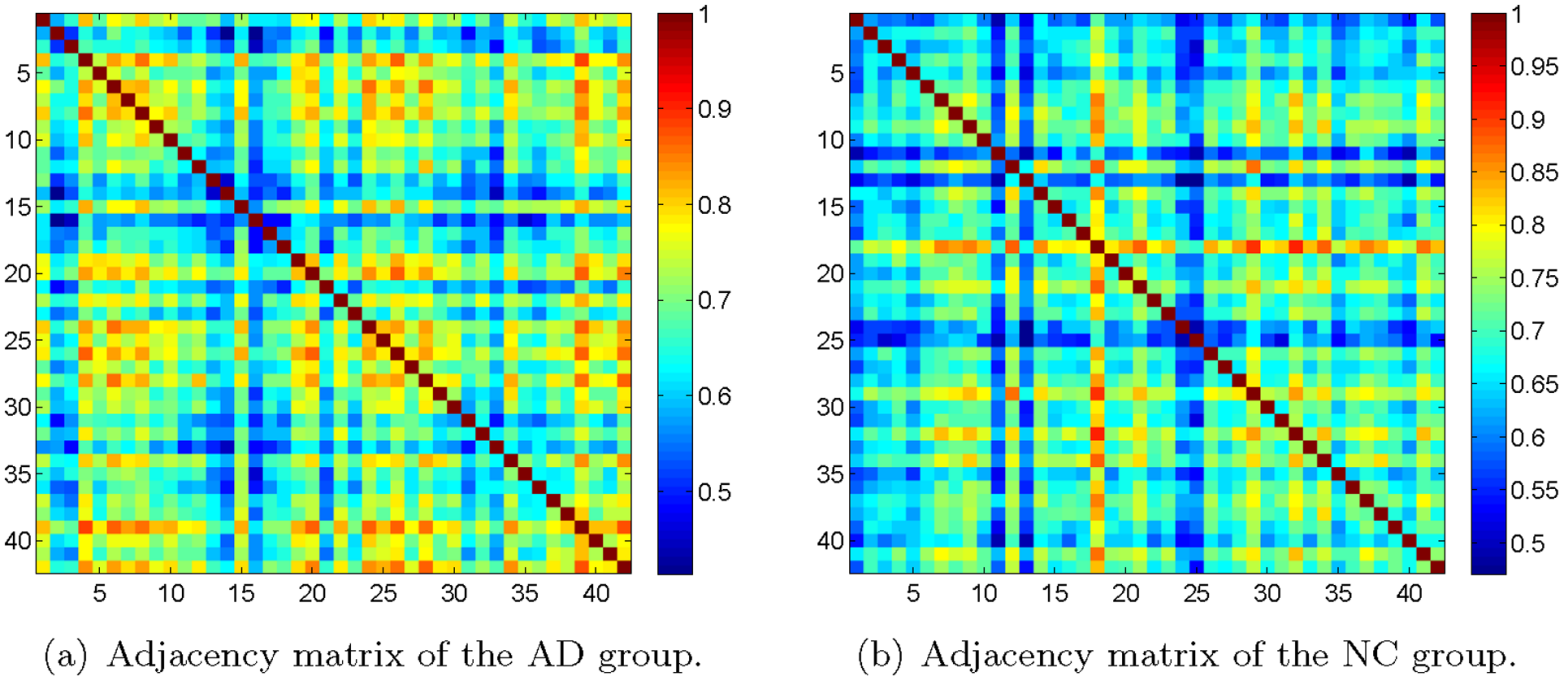
Adjacency matrices obtained by NMI. Adjacency matrices obtained by computing the normalized mutual information between observations of every pair of brain regions, for the AD group (left panel) and the NC group (right panel), respectively.

In what follows, we discuss the networks constructed with NMI. By calculating the NMI, we obtained the adjacency matrices of the AD and NC groups in Fig 9. First of all, from the adjacency matrices, we can not discriminate the block-structure along the diagonal as that appears in the networks learned by the GRM and the graphical Lasso. This is confirmed by calculating the modularity. From Table 4, we observe that the maximized modularity of the network of either the AD group or the NC group is close to zero and significantly less than that of the corresponding network yielded by GRM or GLasso. It indicates that the obtained networks by NMI are almost like random networks, contradicted with the well-accepted findings of the modular structure in brain networks [51].

Moreover, we computed the clustering coefficient and global efficiency of the weighted graphs obtained by NMI. Since the definitions of these two quantities are based on weighted graphs, it allows us to directly analyze the networks without thresholding. As shown in Table 4, the clustering coefficient and global efficiency of the networks of the AD and NC groups are (0.6778, 0.6800) and (0.6816, 0.6832), respectively. Although we observe that the clustering coefficient and global efficiency for the AD group are lower than that of the NC group accordingly, the percentage differences 0.56% and 0.47% are very small.

## Conclusion

We have proposed a GRM to estimate the brain network structure based on neuroimaging data. Our assumption was that the data were smooth signals on a potential graph. The learning procedure was formulated as an optimization of the fit between the graph and the data, with a uniformity level regularization on the connection weights. Both synthetic and real data sets were used to evaluate the proposed method. Results on simulated data indicated that our approach can obtain a very close reconstruction of the ground truth. We then applied the GRM to learn the structural brain connectivity of AD and NC subjects from PiB-PET imaging data.

We believe that the resulting connectivity patterns were easy to interpret and consistent with established knowledge of AD. For either the AD or NC group, our obtained networks were more balanced when compared with the corresponding networks generated from the sample correlation. Moreover, the revealed hub locations matched the cortical hubs given by previous literature using fMRI. We discovered consistent changes between the network of AD group and that of the NC group, by studying the variations of clustering coefficient, global efficiency, maximized modularity, and intra-lobe/inter-lobe connections. The GRM yielded more distinct network structures between the AD and NC groups, when compared with the other methods. Those differentiable network features could potentially help detect AD at elderly or preclinical stages.

## Supporting Information

S1 Appendix Definitions of network metrics. Definitions of the clustering coefficient, global efficiency, and modularity for weighted networks.
(PDF)
