# Supplementary material for: A Spectral Graph Regression Model for Learning Brain Connectivity of Alzheimer’s Disease
Source: PLoS One. 2015 May 29;10(5):e0128136. doi: 10.1371/journal.pone.0128136 (PMC4449104; doi:10.1371/journal.pone.0128136)
Supplement: S1 Appendix — Definitions of the clustering coefficient, global efficiency, and modularity for weighted networks. (PDF) [file pone.0128136.s001.pdf]

# S1 Appendix

For weighted networks, the generalized clustering coefficient $C_{wu}$ [1] and the global efficiency $E_{global}$ [2] are defined as follows

$$C_{wu} = \frac{1}{p} \sum_{i} \frac{1}{D_{ii}(D_{ii}-1)} \sum_{j,k} (W_{ij} W_{ik} W_{jk})^{1/3}, \tag{S1}$$

$$E_{global} = \frac{1}{N(N-1)} \sum_{i \neq j} \frac{1}{l_{ij}}, \tag{S2}$$

where $D_{ii} = \sum_j W_{ij}$ is the degree of vertex $i$; $l_{ij}$ is the shortest weighted path length between vertices $i$ and $j$; $N$ denotes the number of vertices in the network. Notice that the subscript '$wu$' indicates a weighted undirected network.

There are multiple ways of specifying the modularity. One way to define the modularity of a partition of the network into $c$ communities is [3]

$$Q = \sum_{i=1}^{c} (e_{ii} - a_i^2), \tag{S3}$$

where $e_{ii}$ is the fraction of edges with both end vertices in the same community $i$ and $a_i$ is the fraction of ends of edges adjacent to vertices in community $i$. If we define $B_{ij}$ as the difference between $W_{ij}$ and the expected weight between vertices $i$ and $j$ if edge weights are distributed at random, then the modularity can also be viewed as the normalized summation of $B_{ij}$ over all pairs of $i, j$ that are in a same community.

# References


1. Saramäki J, Kivelä M, Onnela JP, Kaski K, Kertesz J. Generalizations of the clustering coefficient to weighted complex networks. Physical Review E. 2007;75(2):027105.

2. Achard S, Bullmore E. Efficiency and cost of economical brain functional networks. PLoS Computational Biology. 2007;3(2):e17.

3. Newman ME. Modularity and community structure in networks. Proceedings of the National Academy of Sciences. 2006;103(23):8577–8582.
